# Demoralization and Associated Factors in Palliative Oncology Outpatients: A Cross‐Sectional Study

**DOI:** 10.1002/pon.70522

**Published:** 2026-06-10

**Authors:** Michael Ljuslin, Vasileios Chytas, Marie‐Estelle Gaignard, Daniel Gorman, Kate Lally, Emanuele Mazzola, Kabir Nigam, Sholevar Roxanne, Zachary Sager, Miryam Yusufov, Sophie Pautex, James Aaron Tulsky, Yvan Beaussant

**Affiliations:** ^1^ Harvard Medical School Boston Massachusetts USA; ^2^ Department of Supportive Oncology Dana‐Farber Cancer Institute Boston Massachusetts USA; ^3^ Division of Palliative Medicine, Department of Rehabilitation and Geriatrics Geneva University Hospitals Geneva Switzerland; ^4^ Faculty of Medicine University of Geneva Geneva Switzerland; ^5^ Department of Psychiatry Service of Liaison Psychiatry and Crisis Intervention Geneva University Hospitals Geneva Switzerland; ^6^ Department of Oncology Swiss Cancer Center Leman Geneva University Hospitals Geneva Switzerland; ^7^ Department of Medicine, Division of Palliative Medicine Brigham and Women's Hospital Boston Massachusetts USA; ^8^ Department of Data Science Dana‐Farber Cancer Institute Boston Massachusetts USA; ^9^ Department of Psychiatry Mclean Hospital Belmont Massachusetts USA

**Keywords:** adjustment disorder, cancer, cross‐sectional studies, demoralization, depression, mood disorders, palliative care, psycho‐oncology, quality of life

## Abstract

**Background:**

Demoralization is common in palliative psycho‐oncology settings. Although its relationship with other psycho‐existential conditions has been scrutinized, its co‐occurrence with adjustment disorder, as measured by the *Adjustment Disorder New Module‐20* (ADNM‐20), remains unexamined.

**Aim:**

Estimate frequency and co‐occurrence of demoralization and adjustment disorder symptom domains among palliative outpatients and examine sociodemographic and psycho‐existential factors.

**Methods:**

A single‐site, cross‐sectional survey at an academic cancer center used self‐reported web‐based screening questionnaires. Analyses included descriptive statistics, association tests, and hierarchical logistic regression models.

**Results:**

Moderate‐to‐severe demoralization symptoms occurred in 43.3% (90% CI: 36.9–49.7) of the 164 outpatients. Among these, co‐occurrence with high‐risk adjustment disorder symptoms reached 77.6% (90% CI: 69.5–85.8) versus 54.9% (90% CI: 45.2–64.6) for moderate‐to‐severe depression symptoms (*p* < 0.001). High‐risk adjustment disorder was also frequent (44.1%, 90% CI: 34.4–53.8.3) and, along with moderate‐to‐severe depression (OR 7.23 and 8.67, *p* < 0.01), was independently associated with moderate‐to‐severe demoralization.

**Conclusions:**

Approximately half of oncology outpatients screened positive for demoralization or adjustment disorder, with substantial co‐occurrence reflecting their conceptually defined shared disruption of adaptive capacities. Demoralization demonstrates incomplete overlap at the symptom‐domain level and appears to capture a distinct existential dimension of distress. This pattern supports consideration of routine screening and further empirical evaluation of its potential diagnostic positioning. Clinician‐administered longitudinal studies incorporating item‐level analyses are warranted to clarify temporal relationships, refine discriminant validity, and further evaluate demoralization as a distinct diagnostic entity. Such work may enhance recognition of existential suffering and inform targeted integrative interventions.

## Key Points

- Nearly half of oncology outpatients screened positive for demoralization, three‐quarters also for adjustment disorder, indicating substantial co‐occurrence.
- Findings suggest shared adaptive disruption, while demoralization remains distinct by indexing impaired meaning‐making
- The observed symptom‐domain non‐redundancy supports consideration of routine screening and further investigation of demoralization's potential diagnostic positioning.

## 1 Background

Despite the high prevalence of demoralization among cancer palliative care outpatients, its association with psycho‐existential distress remains poorly understood. Demoralization, incorporated into the International Classification of Diseases (MB22.2) [1] in 2019, not yet recognized in the Diagnostic and Statistical Manual‐5, encompasses five key factors: loss of meaning and purpose, subjective incompetence, helplessness, leading to hopelessness and dysphoria [2, 3].

This is particularly common in oncology and palliative care settings, where it has been associated with symptom burden [4], heightened mortality awareness [5], and a strong inverse association with quality of life [6, 7, 8, 9]. As death approaches, coping strategies may progressively fail, giving rise to helplessness and hopelessness [2, 4, 10]. A 2023 review of 12,427 oncology patients reported a prevalence of demoralization ranging from 13.5% to 49.4% [4, 6].

Similar to demoralization, Adjustment Disorder (AD) involves difficulties in coping, resulting in distress. In the ICD‐11, AD is defined by two core symptom‐based components: preoccupation with the stressor (e.g., worry, rumination) and failure to adapt (e.g., emotional turmoil, functional impairment) [1]. The construct is unidimensional, focusing on the stressor‐symptom temporal link, and is measured by the *Adjustment Disorder–New Module–20* (ADNM‐20) questionnaire, validated in oncological populations [11, 12].

Despite their conceptual distinctions, demoralization and AD exhibit substantial overlap, particularly in fundamental coping mechanisms intrinsic to both conditions. In contrast, demoralization extends beyond the unidimensional stressor‐symptom framework by integrating an existential dimension. Consequently, some researchers have proposed demoralization as a potential DSM‐5 specifier within the context of AD [6]. Their phenomenological overlap remains insufficiently elucidated highlighting a scientific gap we intend to address [12].

This study aimed to further clarify the clinical phenomenology of demoralization and AD symptom domains, as assessed by the ICD‐11‐derived ADNM‐20, among oncology outpatients receiving palliative care. We hypothesized that demoralization and AD symptom domains would display substantial co‐occurrence within this advanced cancer outpatient cohort.

## 2 Methods

### 2.1 Study Design and Participants

We conducted a cross‐sectional study to examine the frequency and co‐occurrence of psycho‐existential conditions among English‐speaking adults with cancer receiving outpatient palliative care at the Dana‐Farber Cancer Institute. The study was approved by the Harvard Cancer Center Institutional Review Board (Protocol Number 22‐037, approved on May 27, 2021). Results reporting adhered to the *Strengthening the Reporting of Observational Studies in Epidemiology* (STROBE) guidelines [13].

### 2.2 Recruitment & Data Collection

Eligible patients were identified via the clinician's daily schedule screening and invited following consultation by email. Clinicians could exclude patients based on their clinical judgment. Participants were offered a $20 gift card as an optional incentive. Data were collected via the Research Electronic Data Capture (REDCap) web‐based platform after informed, anonymous consent was provided using a 116‐item self‐report battery.

### 2.3 Measures and Sample Size

The primary outcome was demoralization frequency, assessed using the 24‐item, 5‐point Likert‐scale self‐reported *Demoralization Scale I* (DS‐I) instrument. A validated cut‐off of ≥ 30 was used to dichotomize participants into *those with low* versus *moderate‐to‐severe demoralization symptoms* (MSD) [3]. Cronbach's alpha was 0.91, indicating high internal consistency in cancer outpatients.

The target sample size of 178 participants was determined using a similar published cohort frequency of 21% with a margin of error of ± 5% at the 90% confidence level [14]. Secondary outcomes included associations with clinical and sociodemographic factors, particularly a positive ADNM‐20 screen (cut‐off ≥ 48) [12], hereafter referred to as *High‐Risk Adjustment Disorder symptoms* (HR‐AD) [12]. Cronbach's *α* was 0.94 [12].

We administered validated screening instruments for depressive symptoms (Patient Health Questionnaire‐9 [PHQ‐9]) [15] and anxiety (Generalized Anxiety Disorder 7‐item scale [GAD‐7]) [15]. In addition, we used the Primary Care Post‐Traumatic Stress Disorder instrument (PC PTSD) [15] and the Death and Dying Distress Scale (DADDS). Positive screens reflect clinically relevant symptom levels exceeding validated thresholds and do not constitute formal clinical diagnoses, supporting an exploratory and hypothesis‐generating approach [13]. Diagnostic differentiation would require a clinician‐administered structured assessment.

### 2.4 Statistical Analysis

Participants with incomplete DS‐I data were excluded from the analyses performed using R version 4.4.1 (R Foundation for Statistical Computing, Vienna, Austria). Given the exploratory nature of this study, no subgroup, interaction, or sensitivity analyses were planned. For inferential analyses, we adopted a conservative significance threshold (*p <* 0.01) to indicate statistically robust associations [13]. Frequency estimates were reported with 90% confidence intervals to reflect descriptive precision objectives, whereas regression models followed conventional 95% inferential standards.

Clinical variables were tabulated across the two DS‐I categories, and associations were tested using Fisher's exact test.

A hierarchical logistic regression was performed using a dichotomized demoralization index.

Missing data were handled using available case analyses, and multivariable models were based on complete cases.

## 3 Results

### 3.1 Patient Flow and Sample Size

Between April and June 2022, 2420 outpatient visits comprising 1133 unique patients were screened; 889 patients were invited to participate, 220 provided informed consent, and 164 completed the DS‐I, constituting the final analytic sample.

Clinician‐based exclusions were most often related to recent hospitalization, hospice enrollment, or unstable clinical or psychological status, frequently including anxiety. The post‐consent analytic attrition was 25.5% (56/220), primarily due to incomplete DS‐I questionnaires. A participant study flow diagram is provided in the Supplementary Material (Supporting Information S1: Figure S1).

### 3.2 Participant Characteristics

Most respondents identified as female (79.1%) and white (87.7%), were married (65.6%), had completed college or higher education (62.2%), had metastatic disease (68.5%), and were receiving oncological treatment (79.1%). A patient and disease characteristics table is provided in the Supplementary Material (Supporting Information S1: Table S1).

### 3.3 Univariable Association Analysis

In the univariable analysis, several associations were statistically significant. A higher frequency of MSD was found among participants aged ≤ 60 years (70.4%; 90% CI: 61.5–79.3) than among those older (29.6%; 90% CI: 20.7–38.5; *p* = 0.002).

### 3.4 Frequency and Co‐Occurrence Across Symptom Domains

**FIGURE 1:**
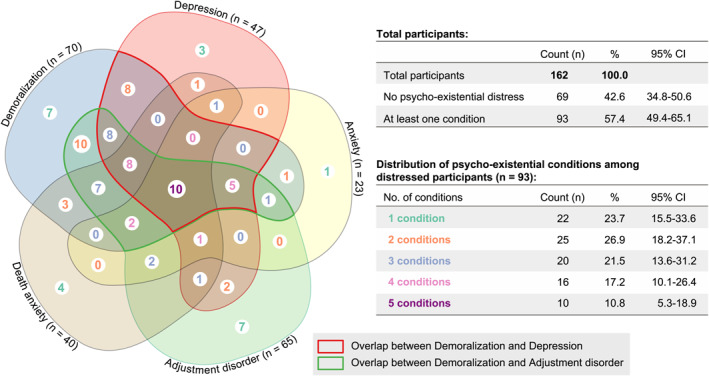
Venn diagram of co‐occurrence of positive symptom screens. This Venn diagram illustrates the intersections of positive self‐reported symptom screens, which were dichotomized at validated clinical cut‐offs (see Table 1 and Supporting Information S1: Table S2). The Venn diagram was based on a complete‐case analytic subsample (*N* = 162) for the included symptom domains after the exclusion of two participants who screened positive for PTSD only. The domains include positive screening for moderate‐to‐severe demoralization symptoms (MSD) (blue), high‐risk adjustment disorder (HR‐AD) (green), moderate‐to‐severe depressive symptoms (red), moderate‐to‐severe death‐related distress or death anxiety (brown), and moderate‐to‐severe generalized anxiety symptoms (yellow). Moderate‐to‐severe demoralization showed the greatest co‐occurrence with high‐risk adjustment disorder (green outline), followed by depressive symptoms (red outline). In conceptual terms, this pattern is compatible with a consistently shared process of adaptation failure between MSD and HR‐AD symptoms, inherently linked to the existential component of loss of meaning. %, percentage; CI, confidence interval; MSD, moderate‐to‐severe demoralization symptoms; n, subgroup; PTSD, Post‐traumatic Stress Disorder.

The proportion of participants who screened positive for MSD was 43.3% (71/164; 90% CI: 36.9–49.7), whereas HR‐AD (ADNM‐20 ≥ 48) was observed in 44.1% (67/152; 90% CI: 37.5–50.7; *n* = 12 missing). Table 1 presents the distribution of participants who exceeded the validated screening thresholds. Participants who met the criteria for any screened condition were significantly more likely to present with positive MSD screening (*p* < 0.001). The co‐occurrence of screened‐positive symptom domains is shown in a Venn diagram (Figure 1).

**TABLE 1:** Co‐occurrence of positive symptom screens between dichotomized demoralization and other psycho‐existential domains.

	Demoralization						Missing	*p*‐value
	Low (*n* = 93) % (*n*)	95% CI:	Moderate‐to‐Severe (*n* = 71) % (*n*)	95% CI:	Total (*N* = 164) % (*n*)	95% CI:		
High‐risk of adjustment disorder^b^	17.6 (15)	10.2–27.4	77.6 (52)	65.8–86.9	44.1 (67)	36.0–52.3	12	< 0.001^g^
Moderate‐to‐severe depression^a^ ^,^ ^c^	8.8 (8)	3.8–16.6	54.9 (39)	42.7–66.8	29.0 (47)	22.2–36.7	2	< 0.001^g^
Post‐traumatic stress disorder^a^ ^,^ ^d^	10.1 (9)	4.3–18.3	46.5 (33)	34.5–58.7	26.2 (42)	19.6–33.8	4	< 0.001^g^
High death and dying distress^e^	11.1 (9)	5.2–20	46.0 (29)	33.4–59.1	26.4 (38)	19.4–34.4	20	< 0.001^g^
Moderate‐to‐severe anxiety^a^ ^,^ ^f^	5.5 (5)	1.8–12.3	26.1 (18)	16.2–38.0	14.4 (23)	9.33–20.8	4	< 0.001^g^

*Note:* Symptoms; all constructs were evaluated using validated self‐report screening instruments; values reflect clinically relevant symptoms above established cut‐offs rather than clinician‐confirmed diagnoses. The cut‐offs applied were as follows:

Abbreviations: %, percentage; AD, Adjustment Disorder; CI, confidence interval; *n*, subgroup; N, total sample; PTSD, Post‐Traumatic Stress Disorder.

^a^ *Demoralization Scale‐I* (DS‐I) ≥ 30 for moderate‐to‐severe demoralization symptoms.

^b^ *Adjustment Disorder–New Module–20* (ADNM‐20) score ≥ 48 for high‐risk AD.

^c^ *Patient Health Questionnaire–9* (PHQ‐9) score ≥ 10 for moderate‐to‐severe depressive symptoms.

^d^ *Primary Care–Post‐traumatic Stress Disorder Screen–5* (PC‐PTSD‐5) score ≥ 3 for probable PTSD symptoms.

^e^ *Death and Dying Distress Scale* (DADDS) score ≥ 47 for moderate‐to‐severe death‐related distress or death anxiety symptoms based on *the Cancer and Living Meaningfully* validation study [16].

^f^ *Generalized Anxiety Disorder–7* (GAD‐7) score ≥ 10 for moderate‐to‐severe anxiety symptoms.

^g^ *p‐*value < 0.01 indicates statistically robust associations.

The co‐occurrence between the MSD and HR‐AD symptom domains was 31.7% (52/164; 90% CI: 25.7–37.7). Among participants with HR‐AD, 77.6% (52/67; 90% CI: 69.2–86.0; *n* = 12 missing) also screened positive for MSD. Conversely, 73.2% (52/71; 90% CI: 64.6–81.9) of participants with MSD also met HR‐AD criteria. Alternatively, 22.4% (15/67; 90% CI: 14.0–30.8) of HR‐AD positive participants did not meet the MSD criteria, while 21.1% (15/71; 90% CI: 13.2–29.1) of MSD positive participants did not meet the HR‐AD criteria.

Overall, 23.8% (39/164; 90% CI: 18.3–29.2) of participants screened positive for both MSD and moderate‐to‐severe depressive symptoms. Among participants screening positive for moderate‐to‐severe depressive symptoms, 83.0% (39/47; 90% CI: 18.3–29.2; *n* = 2 missing) also met MSD criteria. In contrast, only 54.9% (39/71; 90% CI: 45.2–64.6) of participants screening positive for MSD also met criteria for moderate‐to‐severe depressive symptoms, supporting the partial greater independence of demoralization snydome from depressive symptom domains.

No systematic patterns of missingness were identified. Missing data were below 10% across measures and are reported for each estimate.

### 3.5 Factors Associated With Demoralization

In our optimal fit five‐factor hierarchical model of sociodemographic factors, based on an available‐case denominator of 151 participants, no variable was significant.

Using the distress‐variables model, a three‐factor specification based on a complete‐case denominator of 143 participants, achieved the most significant fit. Moderate‐to‐severe depressive symptoms (OR 8.67; 95% CI: 3.06–27.66; *p* < 0.01) and HR‐AD (OR 7.23; 95% CI: 2.88–19.02; *p* < 0.01) were independently associated with MSD (see Supporting Information S1: Table S2).

## 4 Discussion

### 4.1 High Co‐Occurrence With High‐Risk Adjustment Disorder Symptoms

The primary finding of this study is the marked co‐occurrence between MSD and HR‐AD symptoms, which substantially exceeds that observed with depressive symptoms.

Only two studies have explored this co‐occurrence. Vehling et al. reported a 23.5% co‐occurrence in a younger, earlier‐stage cancer population, using an AD DSM‐IV‐derived score [6, 14]. Our more advanced population may reflect a later point in the illness trajectory, at which adaptive exhaustion and existential distress converge.

Bobevski et al. identified a 13.2% subgroup combining adjustment disorder features and moderate demoralization without major mood disturbance in a pooled latent class analysis of early‐ and advanced‐stage cancer patients, interpreted as aligned with adjustment disorder but derived from the DS‐I item domains. A second 10.7% cluster was characterized by severe demoralization and associated with higher rates of mood disorder [8].

Given differences in demographic profiles and diagnostic approaches, direct comparison with the present ICD‐11–based ADNM‐20 study is not feasible [12].

In this study, the autonomous occurrence of MSD in a substantial subset of patients with advanced oncological disorders suggests incomplete phenomenological convergence rather than full construct redundancy.

### 4.2 Frequency of Demoralization

The observed MSD frequency of 43.3% falls within the systematic review's upper range, likely partly reflecting the predominance of advanced‐stage disease [3, 4].

### 4.3 Psycho‐Existential and Psychosocial Factors Associations

Nearly half of the participants with MSD did not meet the criteria for PHQ‐9–positive depressive symptoms, supporting the prior evidence that these constructs are related yet partially independent [4, 5, 17].

Age‐related differences also mirrored prior work, with younger adults showing higher demoralization scores [4, 18]. Such heightened distress may reflect existential awareness of a foreshortened life trajectory; dying young is regularly experienced as incongruent and unjust.

## 5 Clinical and Research Implications

This overlapping pattern suggests a shared disturbance in adaptive functioning that, in this advanced oncological palliative population, is phenomenologically intertwined with existential suffering, as captured by the DS‐I. As hypothesized in previous studies, beyond a certain threshold of cumulative stressors along the illness trajectory, adaptive exhaustion may precipitate existential distress. However, in this advanced oncological outpatient sample, existential distress was observed in a substantial proportion of cases without concurrent AD or mood disorder symptom thresholds, suggesting non‐redundancy across symptom domains.

From a nosological perspective, further multi‐site longitudinal, clinician‐rated studies are needed to determine whether demoralization warrants consideration as a potential diagnostic positioning, rather than a subtype of AD or mood disorder, bearing implications for improving recognition of existential distress while balancing concerns about diagnostic proliferation [6].

Prior studies have shown that demoralization is more strongly associated with suicidal ideation and the desire for hastened death than depression, underscoring its clinical relevance for systematic existential monitoring and timely targeted interventions [14, 17, 19]. Identifying demoralization as a central construct may support the implementation of established interventions, such as Meaning‐Centered Psychotherapy, Dignity Therapy, or Acceptance and Commitment Therapy [20]. It may also provide a conceptual basis for developing existentially and spiritually informed innovations, including emerging psychedelic‐assisted therapies, that may deepen experiences of meaning and support integration of the dying process [21].

## 6 Study Limitations

First, the modest participation rate, together with the overrepresentation of well‐educated White women, suggests self‐selection and volunteer bias, thereby limiting generalizability. Women have been reported to show higher levels of demoralization, which may have inflated the observed frequency [4].

Second, all outcomes relied on self‐report screening instruments, which may introduce recall and desirability biases. These validated tools cannot substitute for clinician‐administered diagnostic evaluations [3, 12]. Furthermore, no factorial or item‐level analyses were conducted to formally assess the latent structure or discriminant validity between constructs. Conclusions regarding partial independence should be interpreted cautiously and limited to symptom‐domain non‐redundancy rather than structural differentiation.

Third, the lack of significant co‐occurrence between MSD and anxiety or death‐related distress may partly reflect a selection bias, as patients with pronounced anxiety were often excluded to minimize the clinical burden. A recent latent class analysis in an oncology population identified anxiety as part of a distinct cluster associated with severe demoralization [8], supporting its potential significance.

Finally, the cross‐sectional design precluded the assessment of temporal changes or causality.

## 7 Conclusion

To the authors' knowledge, this is the first study to examine the co‐occurrence of demoralization and AD using the ICD‐11‐based ADNM‐20 in an outpatient palliative care advanced cancer cohort. MSD was identified in nearly half of the participants, with substantial overlap observed with the HR‐AD criteria and, to a lesser extent, the moderate‐to‐severe depressive symptom threshold.

A meaningful proportion of participants did not meet concurrent screening thresholds across constructs, suggesting only partial overlap between symptom domains. In advanced cancer care, where cumulative illness‐related stressors can profoundly challenge coping capacity and personal meaning, this pattern underscores the clinical relevance of demoralization within the broader psycho‐existential context of palliative oncology. The observed overlap likely reflects common adaptive strain, whereas the non‐overlapping cases suggest dimensions of existential suffering that extend beyond conventional DSM‐5 psychiatric screening categories.

These findings support further longitudinal, clinician‐rated, item‐level research to inform the potential diagnostic positioning of demoralization and facilitate the complementation of symptom‐focused approaches with attention to meaning and dignity.

## Author Contributions

**Michael Ljuslin:** conceptualization, methodology, project administration, investigation, data curation, formal analysis, validation, writing – original draft, writing – review and editing, visualization. **Yvan Beaussant:** supervision, conceptualization, methodology, funding acquisition, data curation, formal analysis, writing – original draft, writing – review and editing, visualization. **James A. Tulsky:** supervision (shared senior authorship), conceptualization, methodology, writing – review and editing. **Sophie Pautex**: supervision, writing – review and editing. **Vasileios Chytas, Marie‐Estelle Gaignard, Daniel Gorman, and Kate Lally:** resources, writing – review and editing. **Kabir Nigam, Zachary Sager, and Roxanne Sholevar:** conceptualization, methodology, writing – review and editing. **Emanuele Mazzola:** data curation, formal analysis, methodology, resources, validation, writing – review and editing. **Miryam Yusufov:** methodology, resources, writing – review and editing. All authors critically revised the manuscript and approved the final version.

## Funding

This work was supported by the Geneva University Hospitals Advanced Development Grant (awarded to Michael Ljuslin) and the Oppenheimer Family Psychosocial Oncology and Palliative Care Research Grant (awarded to Yvan Beaussant).

## Ethics Statement

This study was conducted in accordance with the ethical principles of the Declaration of Helsinki and relevant national and institutional guidelines. Ethical approval was obtained from the Dana‐Farber/Harvard Cancer Center Institutional Review Board (Protocol No. 22‐037) on May 27, 2021. Data were collected anonymously following an introductory consent process using a checkbox. A waiver of documented informed consent was granted. This study adhered to the Committee on Publication Ethics' (COPE) International Standards for Authors, Good Clinical Practice standards, and followed the applicable regulations for research involving human subjects.

## Conflicts of Interest

The authors declare no conflicts of interest.

## Supporting information


Supporting Information S1

## Data Availability

The data that support the findings of this study are available from the corresponding author upon reasonable request. The dataset used in this study is not publicly available. All data were collected anonymously and analyzed in aggregate to ensure participant confidentiality. Data citation: [Dataset] Ljuslin M.; 2025; Demoralization and Associated Factors in Palliative Oncology Outpatients: A Cross‐Sectional Study. Anonymized research dataset; Open Science Framework (OSF); https://osf.io/uzxmn.

## References

[1] J. E. Harrison , S. Weber , R. Jakob , and C. G. Chute , “ICD‐11: An International Classification of Diseases for the Twenty‐First Century,” supplement, BMC Medical Informatics and Decision Making 21, no. S6 (November 2021): 206, 10.1186/s12911-021-01534-6. 34753471 PMC8577172

[2] D. M. Clarke and D. W. Kissane , “Demoralization: Its Phenomenology and Importance,” Australian and New Zealand Journal of Psychiatry 36, no. 6 (December 2002): 733–742, 10.1046/j.1440-1614.2002.01086.x. 12406115

[3] L. Quintero Garzon , A. Hinz , S. Koranyi , and A. Mehnert‐Theuerkauf , “Norm Values and Psychometric Properties of the 24‐Item Demoralization Scale (DS‐I) in a Representative Sample of the German General Population,” Frontiers in Psychology 12 (June 2021): 681977, 10.3389/fpsyg.2021.681977. 34194373 PMC8236510

[4] Y. Wang , H. Sun , Q. Ji , Q. Wu , J. Wei , and P. Zhu , “Prevalence, Associated Factors and Adverse Outcomes of Demoralization in Cancer Patients: A Decade of Systematic Review,” American Journal of Hospice & Palliative Care 40, no. 11 (November 2023): 1216–1230: PubMed PMID: 36718669, 10.1177/10499091231154887. 36718669

[5] S. Robinson , D. W. Kissane , J. Brooker , and S. Burney , “A Systematic Review of the Demoralization Syndrome in Individuals With Progressive Disease and Cancer: A Decade of Research,” Journal of Pain and Symptom Management 49, no. 3 (March 2015): 595–610, 10.1016/j.jpainsymman.2014.07.008. 25131888

[6] L. L. Gan , S. Gong , and D. W. Kissane , “Mental State of Demoralisation Across Diverse Clinical Settings: A Systematic Review, Meta‐Analysis and Proposal for Its Use as a ‘Specifier’ in Mental Illness,” Australian and New Zealand Journal of Psychiatry 56, no. 9 (September 2022): 1104–1129, 10.1177/00048674211060746. 34879712

[7] A. Woźniewicz and F. Cosci , “Clinical Utility of Demoralization: A Systematic Review of the Literature,” Clinical Psychology Review 99 (February 2023): 102227, 10.1016/j.cpr.2022.102227. 36462221

[8] I. Bobevski , D. W. Kissane , S. Vehling , D. P. McKenzie , H. Glaesmer , and A. Mehnert , “Latent Class Analysis Differentiation of Adjustment Disorder and Demoralization, More Severe Depressive and Anxiety Disorders, and Somatic Symptoms in Patients With Cancer,” Psycho‐Oncology 27, no. 11 (November 2018): 2623–2630, 10.1002/pon.4761. 29761579

[9] A. Bovero , M. Opezzo , and V. Tesio , “Relationship Between Demoralization and Quality of Life in End‐Of‐Life Cancer Patients,” Psycho‐Oncology 32, no. 3 (March 2023): 429–437, 10.1002/pon.6095. 36604571

[10] L. Tecuta , E. Tomba , S. Grandi , and G. A. Fava , “Demoralization: A Systematic Review on Its Clinical Characterization,” Psychological Medicine 45, no. 4 (March 2015): 673–691, 10.1017/S0033291714001597. 25032712

[11] Y. Levin , T. Karatzias , M. Shevlin , M. Ben‐Ezra , A. Maercker , and R. Bachem , “The Network Structure of ICD‐11 Adjustment Disorder: A Comparison of Clinical and Nonclinical Samples,” European Psychiatry 65, no. 1 (2022): e43, 10.1192/j.eurpsy.2022.2303. 35903852 PMC9393912

[12] L. Liang , M. Ben‐Ezra , E. W. W. Chan , H. Liu , O. Lavenda , and W. K. Hou , “Psychometric Evaluation of the Adjustment Disorder New Module‐20 (ADNM‐20): A Multi‐Study Analysis,” Journal of Anxiety Disorders 81 (June 2021): 102406, 10.1016/j.janxdis.2021.102406. 33932632

[13] E. V. Elm , D. G. Altman , M. Egger , S. J. Pocock , P. C. Gøtzsche , and J. P. Vandenbroucke , “Strengthening the Reporting of Observational Studies in Epidemiology (STROBE) Statement: Guidelines for Reporting Observational Studies,” BMJ 335, no. 7624 (October 2007): 806–808: AD, 10.1136/bmj.39335.541782.ad. 17947786 PMC2034723

[14] S. Vehling , D. W. Kissane , C. Lo , et al., “The Association of Demoralization With Mental Disorders and Suicidal Ideation in Patients With Cancer,” Cancer 123, no. 17 (September 2017): 3394–3401, 10.1002/cncr.30749. 28472548

[15] D. Anderson , P. J. Vlachostergios , L. Simpson , et al., “A Feasibility Study of Distress Screening With Psychometric Evaluation and Referral of Cancer Patients,” Scientific Reports 15, no. 1 (March 2025): 10397: PubMed PMID: 40140408; PubMed Central PMCID: PMC11947445, 10.1038/s41598-025-94538-5. 40140408 PMC11947445

[16] G. Rodin , C. Lo , A. Rydall , et al., “Managing Cancer and Living Meaningfully (CALM): A Randomized Controlled Trial of a Psychological Intervention for Patients With Advanced Cancer,” Journal of Clinical Oncology 36, no. 23 (August 2018): 2422–2432: PubMed PMID: 29958037; PubMed Central PMCID: PMC6085180, 10.1200/JCO.2017.77.1097. 29958037 PMC6085180

[17] I. Bobevski , D. W. Kissane , S. Vehling , A. Mehnert‐Theuerkauf , M. Belvederi Murri , and L. Grassi , “Demoralisation and Its Link With Depression, Psychological Adjustment and Suicidality Among Cancer Patients: A Network Psychometrics Approach,” Cancer Medicine 11, no. 3 (February 2022): 815–825, 10.1002/cam4.4406. 35122411 PMC8817077

[18] P. L. Tang , H. H. Wang , and F. H. Chou , “A Systematic Review and Meta‐Analysis of Demoralization and Depression in Patients With Cancer,” Psychosomatics 56, no. 6 (November 2015): 634–643, 10.1016/j.psym.2015.06.005. 26411374

[19] A. Costanza , C. Vasileios , J. Ambrosetti , et al., “Demoralization in Suicide: A Systematic Review,” Journal of Psychosomatic Research 157 (June 2022): 110788, 10.1016/j.jpsychores.2022.110788. 35334350

[20] Y. Wang , H. Sun , Q. Ji , J. Wei , and P. Zhu , “Systematic Review of Interventions for Demoralization in Patients With Cancer,” Journal of Nervous and Mental Disease 211, no. 4 (April 2023): 314–326, 10.1097/NMD.0000000000001615. 36975545

[21] S. Schipper , K. Nigam , Y. Schmid , et al., “Psychedelic‐Assisted Therapy for Treating Anxiety, Depression, and Existential Distress in People With Life‐Threatening Diseases,” Cochrane Database of Systematic Reviews 9, no. 9 (September 2024): CD015383: PubMed PMID: 39260823; PubMed Central PMCID: PMC11390284, 10.1002/14651858.CD015383.pub2. 39260823 PMC11390284

