# Supplementary material for: Demoralization and Associated Factors in Palliative Oncology Outpatients: A Cross‐Sectional Study
Source: Psychooncology. 2026 Jun 10;35(6):e70522. doi: 10.1002/pon.70522 (PMC13254010; doi:10.1002/pon.70522)
Supplement: Supplementary file 1 — Supporting Information S1 [file PON-35-e70522-s001.docx]

**Demoralization and Associated Factors in Palliative Oncology Outpatients: A Cross-Sectional Study**

**Supplementary Material**

**Table S1.** **Patient and Disease Characteristics**

|  | n | % |  |  |  |
| --- | --- | --- | --- | --- | --- |
| **Age** |  |  |  |  |  |
| 18-24 | 1 | 0.6 |  |  |  |
| 25-39 | 13 | 8.0 |  |  |  |
| 40-60 | 75 | 46.0 |  |  |  |
| 61-75 | 68 | 41.7 |  |  |  |
| ≥ 75 | 6 | 3.7 |  |  |  |
| *Missing* | *1* |  |  |  |  |
| **Gender** |  |  |  |  |  |
| Female | 129 | 79.1 |  |  |  |
| Male | 34 | 20.9 |  |  |  |
| *Missing* | *1* |  |  |  |  |
| **Race** |  |  |  |  |  |
| Non-white | 20 | 12.3 |  |  |  |
| White | 143 | 87.7 |  |  |  |
| *Missing* | *1* |  |  |  |  |
| **Relationship status** |  |  |  |  |  |
| Married/non-single | 107 | 65.6 |  |  |  |
| Single | 53 | 32.5 |  |  |  |
| Other | 3 | 1.8 |  |  |  |
| *Missing* | *1* |  |  |  |  |
| **Level of education** |  |  |  |  |  |
| 11th grade or less | 4 | 2.4 |  |  |  |
| High school graduate or General Education Development | 20 | 12.2 |  |  |  |
| Two years of college or equivalent | 38 | 23.2 |  |  |  |
| College graduate (Bachelor of Science or Bachelor of Arts) | 40 | 24.4 |  |  |  |
| Master's degree | 46 | 28.0 |  |  |  |
| Doctorate/Medical or Law degree | 16 | 9.8 |  |  |  |
| **Primary cancer** |  |  |  |  |  |
| Solid | 141 | 86.0 |  |  |  |
| Hematological (blood cells) | 9 | 5.5 |  |  |  |
| Other | 14 | 8.5 |  |  |  |
| **Cancer stage** |  |  |  |  |  |
| Localized | 42 | 25.9 |  |  |  |
| Metastatic | 111 | 68.5 |  |  |  |
| Not applicable  (e.g., hematological malignancies) | 9 | 5.6 |  |  |  |
| *Missing* | *2* |  |  |  |  |
| **Diagnosis** |  |  |  |  |  |
| Less than a month ago | 3 | 1.9 |  |  |  |
| Between a month and less than a year ago | 48 | 29.6 |  |  |  |
| Between more than a year and less than five years ago | 73 | 45.1 |  |  |  |
| More than five years ago | 38 | 23.5 |  |  |  |
| *Missing* | *2* |  |  |  |  |
| **Oncological therapy** |  |  |  |  |  |
| Yes | 129 | 79.1 |  |  |  |
| No | 32 | 19.6 |  |  |  |
| I do not know | 2 | 1.2 |  |  |  |
| *Missing* | *1* |  |  |  |  |
| **Time of palliative care follow-up** |  |  |  |  |  |
| < 1 week | 24 | 14.6 |  |  |  |
| ≥ 1 week and < 1 month | 27 | 16.5 |  |  |  |
| ≥ 1 month and < 1 year | 52 | 31.7 |  |  |  |
| ≥ 1 year and < 5 years | 55 | 33.5 |  |  |  |
| ≥ 5 years | 6 | 3.7 |  |  |  |
| **Psycho-oncology follow up** |  |  |  |  |  |
| Social worker | 74 | 45.1 |  |  |  |
| Psychologist | 31 | 18.9 |  |  |  |
| Psychiatrist | 30 | 18.3 |  |  |  |
| Other | 30 | 18.3 |  |  |  |
| No other follow-up | 46 | 28.0 |  |  |  |
| **Spiritual care** |  |  |  |  |  |
| Community | 42 | 25.6 |  |  |  |
| Chaplain | 17 | 10.4 |  |  |  |
| Other | 48 | 29.3 |  |  |  |
| No spiritual care | 85 | 51.8 |  |  |  |
| **Psychotropic medications** |  |  |  |  |  |
| No psychotropic drug | 77 | 47.0 |  |  |  |
| Antidepressant | 69 | 42.1 |  |  |  |
| Anxiolytic | 16 | 9.8 |  |  |  |
| Others | 19 | 11.6 |  |  |  |
| Not sure | 5 | 3.0 |  |  |  |

*Notes:* Data are presented as number (n) and percentage (%). Percentages are based on the available-case denominator for each variable and may not sum to 100% due to rounding.

Abbreviations: N, total sample; n, subgroup; %, percentage.

**Table S2. Odds Ratios for Hierarchical Models Examining Associations Between Demoralization and Other Distress Variables**

| **Dichotomized demoralization index: moderate-to-severe versus low** | | | | | | |
| --- | --- | --- | --- | --- | --- | --- |
|  |  | Models with an increasing number of variables | | | | |
| **Distress ordered** | 1 | 2 | 3 | *95% CI:* | 4 | 5 |
| **Moderate-to-severe depression***^†^****^,^*** *^‡^* | 12.65* | 7.54* | 8.67* | *3.06*–*27.66* | 9.28*^h^* | 7.78* |
| **High-risk of adjustment disorder***^§^* |  | 10.04* | 7.23* | *2.88*–*19.02* | 7.55*^h^* | 7.10* |
| **High death and dying distress***^¶^* |  |  | 2.43 | *0.81*–*7.38* | 2.44 | 1.99 |
| **Moderate-to-severe anxiety***^†^****^,^*** *^††^* |  |  |  |  | 0.71 | 0.52 |
| **Post-traumatic stress disorder***^†, ‡‡^* |  |  |  |  |  | 2.93 |
| Constant | 0.38 | 0.15 | 0.14 |  | 0.14 | 0.12 |
| likelihood ratio test *p*-value | 0 | 0 | <0.001 |  | 0.34 | 0.07 |
| Observations | 162 | 152 | 143 |  | 142 | 142 |
| Log Likelihood | -89.44 | -66.80 | -61.27 |  | -60.82 | -59.21 |
| Akaike Information Criterion | 182.88 | 139.61 | 130.54 |  | 131.64 | 130.41 |

*Notes:* Complete-case multivariable models included between 142 and 162 observations, depending on the predictors included. The retained three-factor model was based on 143 complete cases and selected for superior *p*-values and lower Akaike Information Criterion (AIC) values. Odds ratios (ORs) are presented with standard 95% confidence intervals (CIs).

† Symptoms; All constructs were evaluated with validated self-report screening instruments; values reflect clinically relevant symptoms above established cut-offs rather than clinician-confirmed diagnoses. The cut-offs applied were as follows: *Demoralization Scale-I* ≥30 for moderate-to-severe demoralization symptoms; ‡ *Patient Health Questionnaire–9* (PHQ-9) score ≥10 for moderate-to-severe depressive symptoms; § *Adjustment Disorder–New Module–20* (ADNM-20) score ≥48 for high-risk adjustment disorder; ¶ *Death and Dying Distress Scale* (DADDS) score ≥47 for moderate-to**-**severe death-related distress or death anxiety symptoms; †† *Generalized Anxiety Disorder–7* (GAD-7) score ≥10 for moderate-to-severe anxiety symptoms. ‡‡ *Primary Care–Post-traumatic Stress Disorder Screen–5* (PC-PTSD-5) score ≥3 for probable PTSD symptoms.

* *p*-value < 0.01 indicates statistically robust associations.

Abbreviations: CI, confidence interval; PTSD, Post-traumatic Stress Disorder.

**Figure S1.** **Study Flow Diagram**

**Total office visits evaluated N=2420**

**Patients assessed for eligibility N=1133**

**Excluded on initial record review n=78**

Lack of English proficiency

**IDENTIFICATION**

**Excluded by clinician judgment** **n=166**

Recent/ongoing hospitalization or hospice enrollment 25

Unstable clinical or psychological status 21

Newly referred to palliative care 20

Missed Scheduled visit 10

Severe cognitive impairment 8

No email address available 6

No cancer diagnosis 4

Follow-up period ended 2

Declined participation at visit 1

Other clinical or logistical reasons 69

**Eligible patients invited by email N=889**

**RECRUITMENT**

**Did not enter the study (recruitment non-participation) n=669**

No response 585

Invalid email address 65

Declined participation (opted-out) 19

**Participants who provided informed consent and enrolled in REDCap N=220**

**CONSENT AND DATA ANALYSIS**

**Provided questionnaire data** n=203

- At least one item completed

**Provided analyzable questionnaire data**  180

- At least one questionnaire completed

**Final analytical sample N=164**

- Demoralization Scale I completed

*Note:* Of 2,420 outpatient visits screened, 1,133 patients were assessed for eligibility, and 889 were invited to participate by email. Recruitment-stage non-participation occurred primarily due to non-response, invalid contact information (including undeliverable email addresses), or active refusal. Overall, 24.7% (220/889) of patients invited provided informed consent and enrolled in the REDCap questionnaires (220/889), among whom 20.2% (180/889) completed at least one questionnaire and 18.4% (164/889) completed the *Demoralization Scale* (DS-I) questionnaire, constituting the final analytic sample. Among consented participants, 81.8% (180/220) completed at least one questionnaire, and 74.5% (164/220) completed the Demoralization Scale–I (DS-I), constituting the final analytic sample. Seventy-one participants requested the $20 gift cards offered. “Unstable clinical or psychological status” included frequent anxiety episodes. “Other clinical or logistical reasons” for exclusion included clinician-initiated withdrawal, logistical barriers, or unspecified causes.

Abbreviations: N, total sample; n, subgroup; CI, confidence interval; REDCap, Research Electronic Data Capture.
